# Association of Patients' Characteristics with Acupuncture Treatment Outcomes in Treating Bell's Palsy: Results from a Randomised Controlled Trial

**DOI:** 10.1155/2019/6073484

**Published:** 2019-08-15

**Authors:** Xianjun Xiao, Qianhua Zheng, Yunzhou Shi, Leixiao Zhang, Ling Zhao, Siyuan Zhou, Wei Zhang, Wei Cao, Ying Liu, Ying Li

**Affiliations:** ^1^College of Acupuncture and Massage, Chengdu University of Traditional Chinese Medicine, Chengdu 610075, China; ^2^Rehabilitation Department, The People's Hospital of Jianyang City, Chengdu 641400, China

## Abstract

**Background:**

Acupuncture has been found to be effective for treating Bell's palsy (BP). However, which class of BP patients will have a better response to acupuncture remains uncertain and requires investigation.

**Methods:**

We performed a secondary analysis of a multicenter, randomized, controlled trial. BP patients were randomly divided into five acupuncture treatment groups. The degree of facial nerve recovery was assessed according to the House–Brackmann grading system (HB grade). Grade I was defined as complete recovery (CR), and grades II–VI were defined as incomplete recovery (IR). The relevant patient characteristics were collected and compared between CR and IR groups by univariate and logistic regression analyses.

**Results:**

Eight-hundred twenty-six subjects were analyzed. Among these, 698 (85%) subjects had a good prognosis. No significant difference in the effectiveness of the five treatments was observed (all *P* > 0.05). The likelihood of IR increased by 2.2% with each one-year increase in age (odds ratio (OR) 1.022, 95% confidence interval (CI) 1.005–1.038; *P*=0.009). The likelihood of IR increased by 9% with each kg/m2 increase in BMI (OR 1.090, 95% CI 1.019–1.165; *P*=0.012). The likelihood of IR at the recovery stage was higher than that at the acute stage (OR 7.996, 95% CI 4.570–13.991; *P* < 0.001), and the likelihood of IR of patients with lesions at or above the chorda tympani was higher than that of patients with lesions below the chorda tympani (OR 1.989, 95% CI 1.256–3.150; *P*=0.003). The likelihood of IR increased by 281.7% with each unit increase in the HB grade (OR 2.817, 95% CI 2.113–3.756; *P* < 0.001).

**Conclusions:**

Younger patients at the acute stage of the disease with low BMIs, low initial HB grades, and lesions below the chorda tympani were more likely to respond to acupuncture.

## 1. Introduction

Bell's palsy (BP, idiopathic peripheral facial palsy) is a common disorder caused by the impaired function of nerves that control the movement of facial muscles, thereby affecting facial expressions and communication [1]. The disorder presents as an acute, unilateral, lower motor neuron-type facial weakness with symptoms of postauricular pain, dysgeusia, loss of facial sensations, and hyperacusis [2]. The prognostic outcome of BP is generally favorable, with 71% of subjects recovering in the absence of treatment and 29% of subjects suffering from varying degrees of disability [3]. Furthermore, the sequelae of BP often add to the existing complications of the disease. At present, there is no treatment that is effective for all patients. For this reason, it is important to identify the factors that are likely to increase or decrease the benefits of a given treatment, as this information may improve the overall prognosis [4].

In China, acupuncture is commonly used to treat BP. Acupuncture has been shown to alleviate the symptoms of BP [5] although some patients do not respond to it. Insights have been gained from previously published studies that have investigated why some subjects with chronic pain [4], insomnia [6], or chronic constipation [7] do not respond to acupuncture treatment.

To the best of our knowledge, there is no study on the BP patients that benefit most from acupuncture. This information is important, as it may improve treatment protocols for patients with BP. The aim of this study was to identify the factors contributing to the good prognosis of patients with BP who received acupuncture. Specifically, we performed a secondary analysis of our earlier paper [8] that described a multicenter, randomized, and controlled trial.

## 2. Materials and Methods

### 2.1. Study Design and Patients

Readers are requested to refer to our previously published paper for the detailed study protocol [9]. This earlier paper [8] described a multicenter, randomized, and controlled trial that compared five acupuncture treatments. The study was conducted in three tertiary referral centers in China from December 2007 to August 2009, and it consisted of a 1-week baseline period, 4-week acupuncture treatment period, and 12-week follow-up period. Nine hundred patients were enrolled, and there were 180 patients in each of the five groups. We excluded nine patients, and 65 patients dropped out of the study, with 826 patients (Table 1) remaining in the study until week 16. All of the patients met the predefined diagnostic criteria and received a HB grade [10].

BP was divided into three stages, namely, the acute stage (1–7 days after disease onset), resting stage (8–20 days after disease onset), and recovery stage (21–70 days after disease onset) [9]. The five acupuncture treatment groups, which used similar acupoints, were as follows: (i) manual acupuncture, (ii) manual acupuncture + moxibustion, (iii) manual acupuncture + electroacupuncture, (iv) manual acupuncture + acupuncture along the yangming musculature, and (v) manual acupuncture, regardless of which stage of the disease the patients were in. For groups (i) to (iv), the treatment was adjusted according to the stage of the disease.

### 2.2. Secondary Analysis Design

The primary outcome was facial nerve function measured by HB grade [5, 11]. The HB grade is divided into six grade scores based on 4 standard facial expressions, namely, at rest, raised eyebrows, eyes tightly closed, and showing teeth: grade I indicates normal function and grade VI indicates complete paralysis. Facial nerve recovery was assessed at 12-week follow-up and scored according to the HB grade. Grade I was defined as complete recovery, and grades II–VI were defined as incomplete recovery [5].

We hypothesize that there is an association between the acupuncture response and patient characteristics (i.e., age, gender, body mass index (BMI), occupation, educational level, marital status, side of palsy, nerve lesion site, season of onset, duration of the disease from onset to treatment, history of acupuncture treatment, comorbidity, initial HB grade, and treatment type). The side of palsy was defined as the affected side of the face, and nerve lesions were defined based on clinical manifestations. Muscle twitching, eyelid and/or mouth drooping, and inability to close an eye indicated the presence of a lesion below the chorda tympani, whereas facial weakness, and/or loss of taste, and/or hyperacusis, and/or decreased salivation indicated the presence of a lesion at or above the chorda tympani. The season of onset was defined as the season in which BP occurred. BP was divided into three stages, namely, the acute stage (1–7 days after disease onset), resting stage (8–20 days after disease onset), and recovery stage (21–70 days after disease onset) [9]. The duration of the disease from onset to treatment was defined as the stage in which patients received acupuncture. The initial HB grade was defined as the HB grade received at baseline.

### 2.3. Statistical Analysis

Descriptive statistics were used to analyze the patient characteristics, including age, gender, BMI, and occupation (Table 2). Normally distributed data were expressed as the mean ± standard deviation, whereas nonnormally distributed data were expressed as medians and interquartile ranges. Categorical data were presented as percentages. The prognostic factors of the complete and incomplete recovery groups were analyzed by univariate analysis. For continuous variables, comparisons between the groups were made by Student's *t*-test or Mann–Whitney *U* test. For categorical variables, comparisons between the groups were made by the chi-square test. Before the logistic regression analyses, the variance inflation factor (VIF) was used to detect multicollinearity among the independent variables. Multicollinearity was considered if the VIF for one of variables exceeded 5. Variables that were significant (*P* < 0.1) in univariate analysis was considered as potential candidates for the logistic regression analysis [4]. We added the treatment assignment as a categorical variable to our model and assessed the interactions between the treatment assignment and the candidate variables introduced into the logistic regression model. Backward elimination with a bootstrap approach (using 1,000 bootstrap samples of all the data) was used to select independent variables to be retained in the final model. A two-sided *P* value <0.05 was considered significant. Statistical analysis was performed with SPSS 20.0 software (SPSS, Chicago, IL, USA) and SAS statistical package VERSION 9.4 (SAS Institute, Cary, NC).

## 3. Results

### 3.1. Recovery of BP Patients at Different Stages of the Disease according to the Five Treatments

Eight-hundred twenty-six BP patients were analyzed, of which 698 (85%) were classified as complete recovery. No significant difference in the effectiveness of the five treatments was observed (Table 1). Complete recovery was noted at 4-week follow-up in patients of the manual acupuncture group (76.51% (127/166)), manual acupuncture + moxibustion group (75.15% (124/165)), manual acupuncture + electroacupuncture group (69.05% (116/168)), manual acupuncture + acupuncture along the yangming musculature group (75.00% (120/160)), and nonselective manual acupuncture group (74.85% (125/167)). Complete recovery was noted at 12-week follow-up in patients of the manual acupuncture group (85.54% (143/166)), manual acupuncture + moxibustion group (87.27% (144/165)), manual acupuncture + electroacupuncture group (79.76% (134/168)), manual acupuncture + acupuncture along the yangming musculature group (85.63% (137/160)), and nonselective acupuncture group (85.43% (141/167)). Complete recovery was noted in 74% (612/826) and 85% (698/826) of subjects at 4- and 12-week follow-up, respectively.

### 3.2. Logistic Regression Analysis of Related Factors in Acupuncture Responders

The demographical and clinical characteristics of the complete recovery group are shown in Table 2. Logistic regression analysis with the backward elimination method identified five out of seventeen variables (9 candidate variables and 8 interactions between the candidate variables and treatment assignment) associating significantly with good prognosis. These variables were age, BMI, nerve lesion site, duration of the disease from onset to treatment, and initial HB grade (Table 3).

The likelihood of incomplete recovery increased by 2.2% with each one-year increase in age (odds ratio [OR] 1.022, 95% CI 1.005–1.038; *P*=0.009). The likelihood of incomplete recovery increased by 9% with each kg/m^2^ increase in BMI (OR 1.090, 95% CI 1.019–1.165; *P*=0.012). The likelihood of incomplete recovery of patients at the recovery stage of the disease was higher than that of patients at the acute stage (OR 7.996, 95% CI 4.570–13.991; *P* < 0.001), and the likelihood of incomplete recovery of patients with lesions at or above the chorda tympani was higher than that of patients with lesions below the chorda tympani (OR 1.989, 95% CI 1.256–3.150; *P*=0.003). The likelihood of incomplete recovery increased by 281.7% with each unit increase in the HB grade (OR 2.817, 95% CI 2.113–3.756; *P* < 0.001). Therefore, younger patients at the acute stage of the disease with low BMIs, low initial HB grades, and lesions below the chorda tympani were more likely to respond to acupuncture.

In addition, the interaction terms between treatment assignment and the key baseline factors were not statistically significant. No collinearities were observed among variables in the logistic regression analysis (VIF < 2). After bootstrapping and adjustment for overfitting, the ROC area of the final model was 0.801.

## 4. Discussion

After pooling the data from our earlier paper, we found an association between the acupuncture response and several factors, namely, age, BMI, nerve lesion site, stage of the disease, and initial HB grade. No correlations between acupuncture response and gender, occupation, educational level, marital status, side of palsy, onset season, history of acupuncture, comorbidity, and treatment type were observed.

Studies have reported correlations between the recovery from BP and epidemiologic factors [3, 11–14]. Several factors, such as old age, high BMI, nerve lesion site at or above the chorda tympani, treatment during the recovery stage, and high initial HB grade, had a negative impact on the prognosis after acupuncture treatment for BP.

With regard to the association between the acupuncture response and age, studies have reported that an older age was an indicator of poor prognosis [3, 15] although other studies have described contradictory findings [12, 16, 17], which may be explained by differences in treatment, as steroids and antivirals were used in one study [12]. In another study, however, older patients with chronic functional constipation responded poorly to acupuncture [7], consistent with our results.

In this study, patients with a high BMI were more likely to have a poor prognosis, as obesity may impede the regeneration of peripheral nerves [11]. Obesity is often associated with hyperglycemia and hypertriglyceridemia, which may cause atherosclerosis. Circulatory disorders, such as atherosclerosis, may contribute to facial paralysis [18].

Initial facial nerve function assessment is important in predicting the prognosis of BP patients during the initial examination [19–21]. The prognosis of patients with high initial HB grades, which indicates more severe impairment of facial nerve function, was worse compared with the prognosis of patients with low initial HB grades. Patients with only partial nerve degeneration showed recovery in the first 3 weeks, whereas those with complete nerve degeneration required more time for recovery [3].

The facial nerve consists of a major motor component, which supports the muscles responsible for facial expression and a small sensory branch that transmits taste sensations from the front two-thirds of the tongue via the chorda tympani nerve [22]. Parasympathetic fibers extend to the lacrimal glands via the greater superficial petrosal nerve, and they reach the sublingual and submaxillary glands via the chorda tympani [23]. The facial nerve is long although the region of the lesion can be deduced from the patient's clinical deficits [24]. For example, if the affected region is at or above the chorda tympani, there will be evidence of ipsilateral peripheral facial weakness, lacrimation, salivation, and/or taste deficits. Furthermore, partially reduced or abolished taste sensations and lacrimal function indicated a poor prognosis [3], which is consistent with our results, suggesting that the higher the affected nerve segment, the worse the prognostic outcome. Thus, identifying the affected nerve segment can help to judge the disease severity and prognostic outcome.

The duration of the disease from onset to treatment was also a prognostic factor of BP. The prognosis was worse for patients who received treatment during the recovery stage of the disease than for those who received treatment during the acute stage, which is consistent with the results of a previous study [3]. Although there was a correlation between the acupuncture response and the treatment time, the overall health of the patients also played a major role in the regeneration of axons, as suggested by Ushio and colleagues [20]. Further studies are needed to understand why the prognosis was worse for BP patients that were treated later in the course of the disease, rather than earlier.

Interestingly, there was no correlation between the prognosis and the history of acupuncture, comorbidity, and marital status. In our study, comorbidity was not a prognostic factor of the acupuncture response in BP patients, which was inconsistent with a previous study [12]. This may be explained by the exclusion criteria, as we excluded several comorbidities, including diabetes mellitus, which may or may not have been a prognostic factor for BP [4, 13, 16, 25].

Our analysis was based on a large RCT for BP, and the main intervention was acupuncture, and not a medicinal agent. To appropriately interpret the results of our study, several limitations must be considered. Firstly, the study, though providing analysis of 826 Chinese subjects, was limited in its narrow race scope and generalizability. Secondly, there is no control group was set in the previous study so that the placebo effect could not be eliminated. Thirdly, most of the BP patients in our study were at the acute stage (1–7 days) or the resting stage (8–20 days). Approximately 15% of patients who were admitted to our hospitals were at the recovery stage, and they likely received other treatments, such as medicinal agents or Chinese herbs, prior to enrollment in the study. Regardless, these patients were enrolled in our study if they met the inclusion criteria. Fourthly, we chose five different treatment protocols according to the stage of the disease, leading to a large selection bias in the methods. Further studies are needed to investigate the significance of other treatments on the prognosis of patients. Fifthly, we did not examine other factors, such as expectation values and stimulation parameters that may have influenced the response to acupuncture. These factors should be investigated to better understand the correlation between the acupuncture response and the prognosis of patients.

## 5. Conclusion

Younger patients at the acute stage of the disease with low BMIs, low initial HB grades, and lesions below the chorda tympani were more likely to respond to acupuncture. Modifying the stage of the disease at which acupuncture is administered may enhance the treatment response. Further studies are needed to test this hypothesis.

## Figures and Tables

**Table 1:** HB grades of subjects at different stages of the disease according to the five acupuncture treatments^a^.

HB grade	Treatment^b^					
	A *N* (%)	B *N* (%)	C *N* (%)	D *N* (%)	E *N* (%)	*P* value
Baseline period						
II	16 (9.64)	15 (9.09)	19 (11.31)	16 (10.00)	16 (9.58)	0.5911
III	58 (34.94)	63 (38.18)	61 (36.31)	58 (36.25)	71 (42.51)	
IV	73 (43.98)	67 (40.61)	58 (34.52)	54 (33.75)	58 (34.73)	
V	18 (10.84)	20 (12.12)	28 (16.67)	31 (19.38)	22 (13.17)	
VI	1 (0.60)	0 (0)	2 (1.19)	1 (0.63)	0 (0)	
Total	166	165	168	160	167	
1-week acupuncture						
I	1 (0.60)	2 (1.21)	1 (0.60)	0 (0)	0 (0)	0.2993
II	38 (22.89)	43 (26.06)	38 (22.62)	35 (21.88)	33 (19.76)	
III	77 (46.39)	80 (48.48)	70 (41.67)	71 (44.38)	90 (53.89)	
IV	45 (27.11)	32 (19.39)	46 (27.38)	47 (29.38)	29 (17.37)	
V	5 (3.01)	8 (4.85)	13 (7.74)	7 (4.38)	15 (8.98)	
Total	166	165	168	160	167	
2-week acupuncture						
I	6 (3.61)	8 (4.85)	4 (2.38)	1 (0.63)	3 (1.80)	0.6443
II	73 (43.98)	76 (46.06)	78 (46.43)	73 (45.63)	77 (46.11)	
III	70 (42.17)	58 (35.15)	60 (35.71)	63 (39.38)	63 (37.72)	
IV	16 (9.64)	22 (13.33)	21 (12.50)	21 (13.13)	21 (12.57)	
V	1 (0.60)	1 (0.61)	5 (2.98)	2 (1.25)	3 (1.80)	
Total	166	165	168	160	167	
3-week acupuncture						
I	14 (8.43)	15 (9.09)	14 (8.33)	6 (3.75)	7 (4.19)	0.3617
II	112 (67.47)	95 (57.58)	103 (61.31)	104 (65.00)	111 (66.47)	
III	29 (17.47)	43 (26.06)	39 (23.21)	45 (28.13)	33 (19.76)	
IV	10 (6.02)	12 (7.27)	10 (5.95)	4 (2.50)	16 (9.58)	
V	1 (0.60)	0 (0)	2 (1.19)	1 (0.63)	0 (0)	
Total	166	165	168	160	167	
4-week acupuncture						
I	85 (51.20)	87 (52.73)	87 (51.79)	75 (46.88)	85 (50.90)	0.5646
II	58 (34.94)	54 (32.73)	56 (33.33)	57 (35.63)	50 (29.94)	
III	18 (10.84)	19 (11.52)	17 (10.12)	24 (15.00)	23 (13.77)	
IV	4 (2.41)	5 (3.03)	7 (4.17)	3 (1.88)	9 (5.39)	
V	1 (0.60)	0 (0)	1 (0.60)	1 (0.63)	0 (0)	
Total	166	165	168	160	167	
4-week follow-up						
I	127 (76.51)	124 (75.15)	116 (69.05)	120 (75.00)	125 (74.85)	0.6421
II	28 (16.87)	33 (20.00)	42 (25.00)	30 (18.75)	29 (17.37)	
III	10 (6.02)	8 (4.85)	8 (4.76)	9 (5.63)	11 (6.59)	
IV	0 (0)	0 (0)	2 (1.19)	1 (0.63)	2 (1.20)	
V	1 (0.60)	0 (0)	0 (0)	0 (0)	0 (0)	
Total	166	165	168	160	167	
12-week follow-up						
I	142 (85.54)	144 (87.27)	134 (79.76)	137 (85.63)	141 (84.43)	0.3999
II	17 (10.24)	18 (10.91)	28 (16.67)	18 (11.25)	20 (11.98)	
III	6 (3.61)	3 (1.82)	5 (2.98)	4 (2.50)	6 (3.59)	
IV	0 (0)	0 (0)	1 (0.60)	1 (0.63)	0 (0)	
V	1 (0.60)	0 (0)	0 (0)	0 (0)	0 (0)	
Total	166	165	168	160	167	

^a^Data are expressed as the number of patients (%), unless otherwise indicated. ^b^A indicates manual acupuncture, B indicates manual acupuncture + moxibustion, C indicates manual acupuncture + electroacupuncture, D indicates manual acupuncture + acupuncture along the yangming musculature, and E indicates manual acupuncture, regardless of which stage of the disease the patients were in.

**Table 2:** Demographical and clinical characteristics of the subjects^a^.

Characteristic	Complete recovery (*N* = 698)	Incomplete recovery (*N* = 128)	Total (*N* = 826)	*P* value
Age	39.35 (14.03)	45.06 (14.47)	40.23 (14.24)	<0.001
Gender				
Male	367 (52.6)	76 (59.4)	443 (53.6)	0.156
Female	331 (47.4)	52 (40.6)	383 (46.4)	
BMI	23.48 (3.22)	24.57 (3.77)	23.65 (3.33)	0.002
Occupation				
White-collar	312 (44.7)	53 (41.4)	365 (44.2)	0.203
Worker or farmer	161 (23.1)	27 (21.1)	188 (22.8)	
Student	70 (10.0)	9 (7.0)	79 (9.6)	
Retired or not working	155 (22.2)	39 (30.5)	194 (23.5)	
Educational level				
Nonuniversity degree	372 (53.3)	77 (60.2)	449 (54.4)	0.152
University or postgraduate degree	326 (46.7)	51 (39.8)	377 (45.6)	
Marital status				
Married	522 (74.8)	108 (84.4)	630 (76.3)	0.019
Not married	176 (25.2)	20 (15.6)	196 (23.7)	
Side of palsy				
Left	347 (49.7)	64 (50.0)	411 (49.8)	0.952
Right	351 (50.3)	64 (50.0)	415 (50.2)	
Nerve lesion site				
Below the chorda tympani	458 (65.6)	61 (47.7)	519 (62.8)	<0.001
At or above the chorda tympani	240 (34.4)	67 (52.3)	307 (37.2)	
Onset season				
Spring	178 (25.5)	38 (29.7)	216 (26.2)	0.419
Summer	205 (29.4)	29 (22.7)	234 (28.3)	
Autumn	129 (18.5)	23 (18.0)	152 (18.4)	
Winter	186 (26.6)	38 (29.7)	224 (27.1)	
Duration of the disease from onset to treatment				
Acute stage	359 (51.4)	50 (39.1)	409 (49.5)	<0.001
Resting stage	262 (37.5)	25 (19.5)	287 (34.7)	
Recovery stage	77 (11.0)	53 (41.4)	130 (15.7)	
History of acupuncture				
No	412 (59.0)	42 (32.8)	454 (55.0)	<0.001
Yes	286 (41.0)	86 (67.2)	372 (45.0)	
Comorbidity				
No	651 (93.3)	112 (87.5)	763 (92.4)	0.024
Yes	47 (6.7)	16 (12.5)	63 (7.6)	
Initial HB grade^c^	3 (3-4)	4 (4-5)	4 (3-4)	<0.001
Type of treatment^d^				
A	142 (20.3)	24 (18.8)	166 (20.1)	0.387
B	144 (20.6)	21 (16.4)	165 (20.0)	
C	134 (19.2)	34 (26.6)	168 (20.3)	
D	137 (19.6)	23 (18.0)	160 (19.4)	
E	141 (20.2)	26 (20.3)	167 (20.2)	

BMI, body mass index. ^a^Data are expressed as the number of patients (%), unless otherwise indicated. ^b^A threshold of *P* < 0.10 was used to select the variables for the logistic regression analysis. ^c^Initial HB grades were expressed as medians and interquartile ranges. ^d^A indicates manual acupuncture, B indicates manual acupuncture + moxibustion, C indicates manual acupuncture + electroacupuncture, D indicates manual acupuncture + acupuncture along the yangming musculature, and E indicates manual acupuncture, regardless of which stage of the disease the patients were in.

**Table 3:** Backward logistic regression with the bootstrap method.

Variable	*B*	SE	*P* value	Odds ratio (95% CI)^a^
Age	0.021	0.008	0.009	1.022 (1.005–1.038)
BMI	0.086	0.034	0.012	1.090 (1.019–1.165)
Nerve lesion site	0.688	0.235	0.003	1.989 (1.256–3.150)
Duration of disease from onset to treatment				
Acute stage			<0.001	
Resting stage	−0.372	0.292	0.082	0.690 (0.389–1.220)
Recovery stage	2.079	0.286	<0.001	7.996 (4.570–13.991)
Initial HB grade	1.036	0.147	<0.001	2.817 (2.113–3.756)
Intercept	−9.254	1.170	<0.001	

BMI, body mass index. ^a^Regression coefficient and corresponding odds ratio after bootstrapping (i.e., adjusted for overfitting).

## Data Availability

The data used to support the findings of this study are included within the article.
